# Computational Modeling of Interactions between Multiple Myeloma and the Bone Microenvironment

**DOI:** 10.1371/journal.pone.0027494

**Published:** 2011-11-08

**Authors:** Yan Wang, Peter Pivonka, Pascal R. Buenzli, David W. Smith, Colin R. Dunstan

**Affiliations:** 1 Department of Infrastructure Engineering, School of Engineering, University of Melbourne, Melbourne, Victoria, Australia; 2 Faculty of Engineering, Computing and Mathematics, University of Western Australia, Perth, Western Australia, Australia; 3 Department of Biomedical Engineering, School of Engineering, University of Sydney, Sydney, New South Wales, Australia; University of Georgia, United States of America

## Abstract

Multiple Myeloma (MM) is a B-cell malignancy that is characterized by osteolytic bone lesions. It has been postulated that positive feedback loops in the interactions between MM cells and the bone microenvironment form reinforcing ‘vicious cycles’, resulting in more bone resorption and MM cell population growth in the bone microenvironment. Despite many identified MM-bone interactions, the combined effect of these interactions and their relative importance are unknown. In this paper, we develop a computational model of MM-bone interactions and clarify whether the intercellular signaling mechanisms implemented in this model appropriately drive MM disease progression. This new computational model is based on the previous bone remodeling model of Pivonka et al. [1], and explicitly considers IL-6 and MM-BMSC (bone marrow stromal cell) adhesion related pathways, leading to formation of two positive feedback cycles in this model. The progression of MM disease is simulated numerically, from normal bone physiology to a well established MM disease state. Our simulations are consistent with known behaviors and data reported for both normal bone physiology and for MM disease. The model results suggest that the two positive feedback cycles identified for this model are sufficient to jointly drive the MM disease progression. Furthermore, quantitative analysis performed on the two positive feedback cycles clarifies the relative importance of the two positive feedback cycles, and identifies the dominant processes that govern the behavior of the two positive feedback cycles. Using our proposed quantitative criteria, we identify which of the positive feedback cycles in this model may be considered to be ‘vicious cycles’. Finally, key points at which to block the positive feedback cycles in MM-bone interactions are identified, suggesting potential drug targets.

## Introduction

Multiple Myeloma (MM) is a B-cell malignancy associated with high morbidity and short survival duration post-diagnosis. 60–70% of MM patients have bone involvement at the time of diagnosis (60% of them with bone pain and 25% of them with bone fracture), and 90% of MM patients will develop bone lesions during the course of the disease [2]–[5]. MM can be associated with a systematic thinning of bone or with the formation of focal osteolytic lesions [6]. The bone lesions result in osteopenia and pathologic fractures (i.e., compression fractures of the spine), which significantly impact on patient morbidity, performance status (including immobility, loss of independence and loss of dignity) and survival duration [3].

Bone is a dynamic tissue that undergoes remodeling in adults, periodically being resorbed by osteoclasts followed by new bone formation by osteoblasts. Coordinated coupling between osteoclast and osteoblast activity is necessary to maintain the balance between bone resorption and bone formation in adults [7], [8]. However, coordination between osteoclasts and osteoblasts is dysregulated in several disease, such as osteoporosis [9] and Paget's disease [10], resulting in an imbalance between bone resorption and bone formation.

In patients with MM, the imbalance between bone resorption and formation occurs through increased osteoclast activity and a lesser increase in osteoblast activity, leading to net bone destruction [5]. The bone loss is often focal and significant, and may lead to the collapse of vertebrae or the breakage of long bones. MM cells cause bone loss through simultaneously promoting osteoclast activity and inhibiting osteoblast activity by secreting various soluble growth factors and cytokines, and by modifying cell-cell adhesion. In addition, growth factors released by bone resorption together with altered cell-cell adhesion facilitate the proliferation of MM cells [5], [11]. It has been postulated that positive feedback loops in the interactions between MM cells and the bone microenvironment form reinforcing ‘vicious cycle(s)’ [12], [13], resulting in elevated bone resorption, which in turn, is then coupled with enhanced MM cell population growth in the bone marrow cavity.

Within the past two decades, a number of prospective components and interactions involved in MM-bone positive feedback cycles have been identified through experiments. Based on these experimental observations, much effort has been made by biologists to integrate the known components and interactions, leading to a few candidate conceptual models of MM-bone positive feedback cycles [5], [14]–[16]. Despite advances in a systematic representation of MM-bone interactions, the dynamics of these interactions and their relative importance are unknown. The issues can be addressed by computational modeling, as it can provide systematic and quantitative insights into MM-bone feedback loops and the way these cycles may interact to cause bone destruction.

The computational modeling of MM-bone interactions involves trade-offs. While a more complete model may be more accurate, waiting until everything is known about the system is not practical. On the other hand, including everything that is currently known may lead to a computational model that is impractical because many unmeasured parameters would have to be estimated. Further, the additional model complexity may result in little gain in understanding. A balance is required between model simplicity and complexity to develop a realistic model that can help address significant questions as to the origin and management of MM-induced osteolysis. Here a key question arises: can the most important mechanisms identified by biologists appropriately drive and explain the MM disease evolution? In terms of a new and tentative computational model of MM-induced osteolysis, it is clearly desirable to include only the most important mechanisms in MM-induced osteolysis.

In comparison to the growing numbers of components and interactions identified by biologists to date, there has been very little investigation of the dynamics of the interplays of these interactions by mathematical/computational modelers. To our knowledge, Ayati et al. [17] recently developed the only mathematical model investigating the dynamics of the MM-bone vicious cycle. In the case of untreated MM, the mathematical model appears to capture some qualitative features of MM disease progress (i.e., an increase in MM-cell density and a decrease in bone volume) in basic multi-cellular units (BMUs) of trabecular bone. However, molecular based cell-cell signaling pathways have not been explicitly modeled, but rather are abstracted into three phenomenological pathways (MM cells inhibit osteoblasts, MM cells increase osteoclasts and bone resorption stimulates tumor growth). For this reason, there is no clear connection between model parameters and the bone physiology (or the MM pathology).

In this paper, we develop a computational model involving feedback cycles between MM and bone cells, and clarify whether the most important cell-cell signaling implemented in this model appropriately drive MM disease progression. The interactions between MM cells and the bone microenvironment have to be properly represented in the model, and then the dynamics of the MM-bone interactions have to be investigated to test whether this model captures major features of MM disease progression. Consequently, two most important tasks are required to develop a suitable model of MM-bone interactions: (i) selecting the most important mechanisms driving MM disease progression; and (ii) parameterizing these mechanisms using chemical and physical model principles informed by biological data. At the same time the proposed computational model is to be kept as simple as possible.

This computational model is based on a previous computational model of bone remodeling (in the absence of MM cells) developed by Pivonka et al. [1], [18]. While Pivonka et al. 's model [1] already explicitly considers several regulatory factors together with bone cells to describe the couplings between bone resorption and bone formation, further bone regulatory factors believed to be dysregulated during MM disease progression need to be incorporated into this bone model. By explicitly considering interleukin-6 (IL-6) and multiple myeloma-bone marrow stromal cell (MM-BMSC) adhesion related pathways, a new tentative MM-bone model is developed, and two positive feedback cycles in MM-bone interactions can then be identified in this model. The MM-bone model is a system of ordinary differential equations (ODEs) that are solved by numerical integration. The parameters of this model are estimated based on reported values in the literature, and when required, from best-fit parameter estimates from a least-square optimization criterion. The dynamics of MM and bone cells predicted by this model are in accord with biological and clinical observations, both in the normal and disease states. The qualitative and quantitative comparison of dynamic simulations with features of the MM disease progression (i.e., increase in the density of bone cells, in the density of MM cells, and in the concentrations of IL-6 and receptor activator of nuclear factor-κB ligand (RANKL), together with decrease in the concentration of osteoprotegerin (OPG), and decrease in the bone volume) shows that the proposed computational model appropriately reflects MM disease progression. In particular, two positive feedback cycles identified in the computational model are sufficient to jointly drive MM disease progression.

With two positive feedback cycles identified, the relative importance of each cycle is not completely clear. While the terminology ‘vicious cycle’ is commonly used in the biological/cancer literature to identify positive feedback loops between the cancer cells and their microenvironment, it is not usually given a quantitative definition. In this paper, quantitative analysis is performed based on comparing total changes of MM-cell density and bone volume over time, when both positive feedback cycles are intact and when either one or the other, or both, of the positive feedback cycles are disabled (i.e., blocked). Using our proposed quantitative criteria, the relative contribution of the two positive feedback cycles is clarified and ‘vicious cycles’ identified. Furthermore, our analysis identifies key regulation molecules that if blocked, would inhibit the positive feedback cycles in MM-bone interactions, thereby suggesting possible drug targets appropriate for either the alleviation of MM-tumor burden or the improvement in MM-induced bone lesions.

The paper is organized as follows. In Section Methods, the MM-bone model structure is described. In Section Results, the governing equations of the (MM-free) bone model and MM-bone model are developed. The progression of MM disease is simulated numerically from normal bone physiology in the absence of MM cells to a well established MM disease state in the presence of MM cells. Simulations are qualitatively and quantitatively compared with clinical observations for normal bone physiology and for MM disease. In Section Discussion, quantitative analysis is performed on the positive feedback cycles that are identified and validated in the MM-bone model.

## Methods

### 2.1 The structure of the MM-bone model

Before positive feedback cycles in the interactions between MM cells and the bone microenvironment can be investigated, the bone microenvironment has to be well understood, as it is a complex system in its own right. Two bone homeostasis models [1], [19], which incorporate parathyroid hormone (PTH), RANKL/OPG/RANK pathway and transforming growth factor β (TGF-β) regulatory couplings between the osteoblast lineage cells and osteoclast lineage cells, were proposed to drive the dynamics of bone cells through the underlying molecular mechanisms. For the model perturbations investigated in Lemaire et al. [19] (e.g., adding external bone cells into the bone model), there is qualitative agreement between experimental and clinical observations, suggesting that the molecular mechanisms included in the model capture key couplings between the osteoblastic lineage and the osteoclastic lineage. Because the Pivonka et al. model [1] is derived from the Lemaire et al. model [19], the two bone models are similar in their behavior. However, one important difference is that while it is assumed that OPG is secreted from osteoblast precursors and RANKL is expressed on active osteoblasts in Lemaire et al. [19], in Pivonka et al. [1] these assumptions are reversed (i.e., RANKL is expressed on osteoblast precursors and OPG is secreted by active osteoblasts). There is extensive biological evidence that supports the Pivonka et al. model [1], and it clearly makes sense at the level of the BMU [20]. As a result, our attempt to model MM-bone interactions is based on the bone model of Pivonka et al. [1].

**Figure 1:**
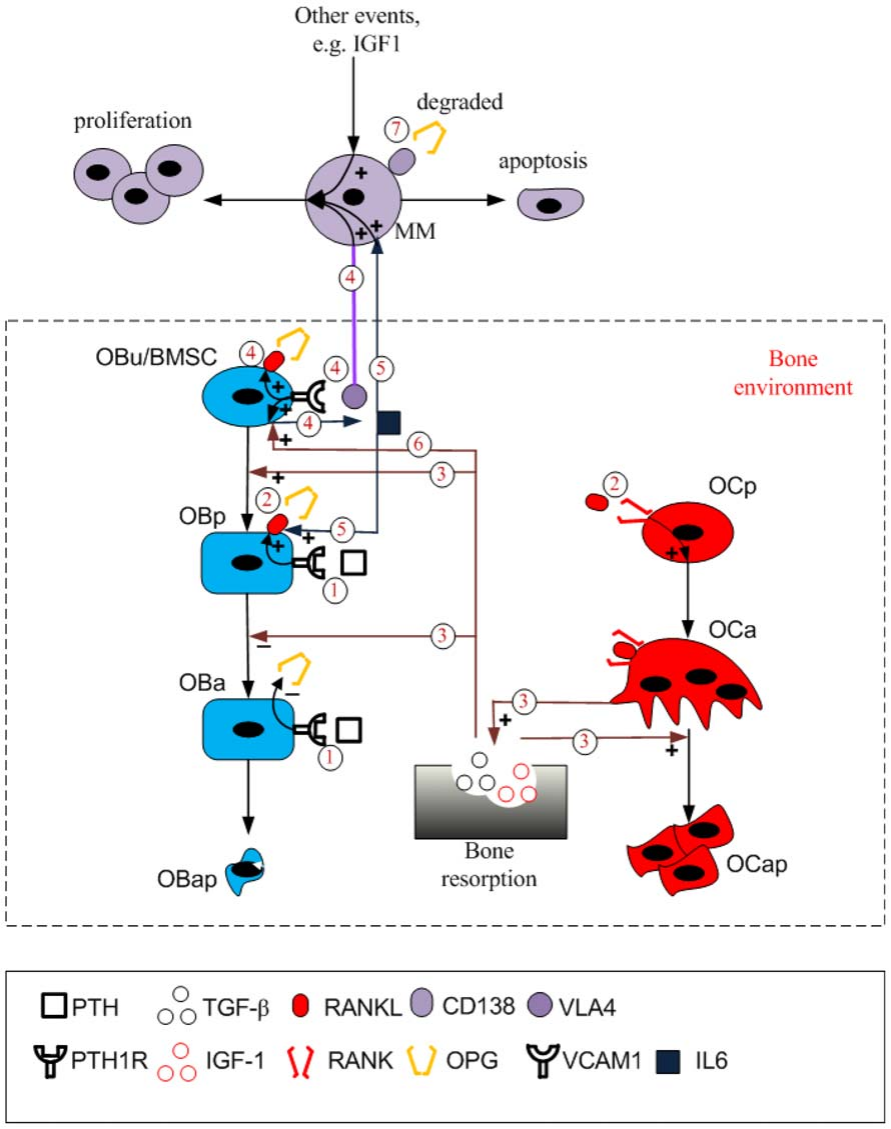
Schematic of the MM-bone model structure. Regulation mechanism 1: PTH stimulates RANKL expression on the surface of osteoblast precursors while inhibiting OPG secretion by active osteoblasts. Regulation mechanism 2: RANKL binds to RANK, which promotes the differentiation of osteoclast precursors, while OPG inhibits the RANKL-RANK binding. Regulation mechanism 3: Bone resorption released TGF-β stimulates uncommitted-osteoblast differentiation, inhibits osteoblast-precursor differentiation and facilitates the apoptosis of active osteoclasts. Regulation mechanism 4: MM cells adhere to BMSC, enabling IL-6 secretion by BMSC, RANKL expression on the surface of BMSC and MM-cell proliferation. Regulation mechanism 5: IL-6 facilitates MM-cell proliferation and stimulates RANKL expression on the surface of osteoblast precursors. Regulation mechanism 6: bone resorption released TGF-β stimulates IL-6 production by BMSC. Regulation mechanism 7: OPG is internalized and degraded by MM cells.

Figure 1 illustrates the structure of this bone model, and the interactions between cells of the osteoblastic lineage and osteoclastic lineage are highlighted by regulation mechanisms 1, 2 and 3. As described in Table 1, PTH stimulates RANKL expression on the surface of osteoblast precursor cells (OB_p_) while PTH inhibits OPG secretion by active osteoblasts (OB_a_). RANKL binds to receptor activator of nuclear factor-κB (RANK) on the surface of osteoclast precursors (OC_p_) triggering the differentiation of osteoclast precursors into active osteoclasts (OC_a_), which is inhibited by OPG due to its competitive binding to RANKL. The relative RANKL and OPG concentration controls osteoclast differentiation and number. In addition, active osteoclasts resorb bone leading to TGF-β being released into the bone microenvironment. The released TGF-β has various actions, including stimulating the differentiation of uncommitted osteoblasts (OB_u_), inhibiting the differentiation of osteoblast precursors and facilitating the apoptosis of active osteoclasts.

**Table 1:** The Description of regulation mechanisms involved in the MM-bone model.

models	Mechanisms	description
Bone model	Regulation mechanism 1	PTH stimulates RANKL expression on the surface of osteoblast precursors, while inhibiting OPG secretion by active osteoblasts.
	Regulation mechanism 2	RANKL binds to RANK, which promotes osteoclast precursor differentiation, while OPG inhibits RANKL-RANK binding.
	Regulation mechanism 3	Bone resorption released TGF-β stimulates uncommitted-osteoblast differentiation, inhibits osteoblast-precursor differentiation and facilitates apoptosis of active osteoclasts.
MM-bone model	Regulation mechanism 4	Adhesion of MM cells to BMSC induces the proliferation of MM cells, production of IL-6 by BMSC and expression of RANKL on the surface of BMSC.
	Regulation mechanism 5	IL-6 facilitates MM-cell proliferation and RANKL expression on the surface of osteoblast precursors.
	Regulation mechanism 6	Bone resorption released TGF-β stimulates IL-6 production by BMSC.
	Regulation mechanism 7	OPG is internalized and degraded by MM cells.

Taking the bone cell population model of Pivonka et al. [1], we first extend this bone model by incorporating regulatory factors that are important for MM in the context of the bone microenvironment (see Section 3.1.1), and further develop the extended bone model by incorporating MM cells and the most important intercellular interactions between MM cells and the bone microenvironment (see Section 3.2.1). Figure 1 illustrates the proposed structure of the MM-bone model. The interactions between MM cells and bone cells are highlighted by regulation mechanisms 4, 5, 6 and 7. These are briefly described in Table 1. Each mechanism and its biological justification are now discussed in turn.

Bone marrow stromal cells (BMSC) are considered as early progenitors derived from mesenchymal stem cells (MSC) and have potential to commit to various mesenchymal cell lineages (including the osteoblast cell lineage) [21], [22]. Accordingly, BMSCs are modeled as uncommitted osteoblasts (OB_u_) rather than osteoblast precursors (OB_p_) in the MM-bone model. Several known conceptual models [14], [15] indicate that MM cells adhere to BMSCs mediated by the adhesion molecules such as very-late antigen 4 (VLA-4) expressed on the surface of MM cells, and vascular cell adhesion molecule 1 (VCAM-1) expressed on the surface of BMSCs. The MM-BMSC adhesion appears to play a number of important roles in MM-bone positive feedback cycles. For example, MM-BMSC adhesion induces MM-cell proliferation through activation of phosphatidylinositol 3-kinase (PI-3K), mitogen-activated protein kinase (MAPK) and nuclear factor-κB (NF-κB) pathways in MM cells [14], [23]. Importantly, NF-κB activation in BMSC induces increased production of IL-6 by BMSCs [14]. Furthermore, MM-BMSC adhesion increases production of RANKL by BMSCs [14]. All these aspects are captured in the MM-bone model and are highlighted in the diagram by regulation mechanism 4.

IL-6 is an ‘osteoclastogenic factor’ that in the bone microenvironment is only produced by cells of the osteoblastic lineage [24], [25]. TGF-β released from the bone matrix during resorption, also stimulates IL-6 secretion by BMSCs through the activation of NF-κB pathway [14], [26]. IL-6 in turn stimulates RANKL production by osteoblast precursors through the STAT3-dependent pathway (while PTH stimulates RANKL production through the PKA pathway) [27]. The IL-6 concentrations are usually small in the normal bone microenvironment, and so are thought not to exert significant effects on osteoclast activity under conditions of normal bone physiology [28]–[30]. However in patients with MM, IL-6 does become significant in the regulation of bone cells [31]. IL-6 is produced in significant quantities by BMSCs in response to MM-BMSC adhesion and activation of the NF-κB signaling pathway [14], [32]–[34]. More specifically, both TGF-β and MM-BMSC adhesion regulate IL-6 secretion by BMSC, and the effect on both regulatory pathways is synergistic [35], leading to substantially increased IL-6 concentrations in the context of MM.

IL-6 is also known to be one of the most important factors stimulating MM-cell proliferation [23]. IL-6 stimulates (via the triple complex IL-6/gp130/IL-6R) the activation of PI-3K, MAPK and NF-κB signaling pathways, which allow MM cells to proliferate and resist the induction of apoptosis by conventional therapeutics such as dexamethasone [23]. It is noted that these down-stream signaling pathways are also triggered by the MM-BMSC adhesion complex [14], [36], [37]. The roles of IL-6 on MM-cell proliferation and osteoclast activity, as well as production of IL-6 in patients with MM, are highlighted in the diagram by regulation mechanisms 5 and 6 respectively.

Osteoclast activity is directly controlled by the RANKL/OPG/RANK pathway. Further RANKL is produced by osteoblast precursors in response to PTH and IL-6 stimulation [27]. Additional RANKL is produced by BMSCs in response to MM-BMSC adhesion [14]. On the other hand, OPG is internalized and degraded by MM cells [38], tending to reduced local OPG concentrations. The increase in RANKL and decrease in OPG leads to an elevated RANKL/OPG ratio and so increased osteoclast activation. This is highlighted in the diagram by regulation mechanisms 4 and 7 respectively.

MM cells are eliminated too. For example, the apoptosis of MM cells occurs due to the actions of T cells [39], [40], but this apoptosis may be inhibited by the action of TGF-β on T cells [41]. However, for the purpose of simplifying our model, the apoptosis rate of MM cells is assumed constant. More detailed regulations of anti-apoptosis of MM cells will be considered in future models.

In addition to IL-6, other soluble factors also contribute to MM-cell population growth and increased bone resorption although their contributions appear to be less than those of IL-6. For example insulin-like growth factor 1 (IGF-1), which is released from the bone matrix during bone resorption, stimulates MM-cell proliferation and survival [23]. Vascular endothelial growth factor (VEGF) is produced by MM cells and stimulates the growth of blood vessels which supports MM cell growth [42], leading to a positive autocrine feedback loop. Macrophage inflammatory protein-1α (MIP-1α) is secreted by MM cells [43] and activates VLA-4 on the surface of MM cells, which enhances MM-BMSC adhesion mediated by VCAM-1 binding to VLA-4 binding [44], [45]. The enhanced MM-BMSC adhesion stimulates IL-6 and RANKL production by BMSCs, suggesting that MIP-1α increases bone resorption by RANKL-mediated pathways [5]. The enhanced MM-BMSC adhesion also induces MM cell proliferation due to increased IL-6 concentrations, which in turn promotes secretion of MIP-1α by MM cells and forms a positive feedback cycle [46]. Given our goal for creating a simple computational model by incorporating representative known factors rather than all known factors, in this computational model all these soluble factors (including IGF-1, VEGF and MIP-1α) are considered to have much smaller effects on MM-cell proliferation than those triggered by IL-6. We acknowledge that this assumption is a potential shortcoming of this computational model.

In patients with MM, it has also been proposed that Wnt signaling is blocked by Dickkopf-1 (DKK-1) [47] and by secreted frizzled-related protein-2 (sFRP-2) [48], which are both secreted by MM cells [49], [50], leading to the inhibition of bone formation in MM disease. However, the precise mechanisms by which DKK-1 and sFRP-2 regulate the osteoblast functions remain to be determined [6], [12]. In the MM-bone model presented here we do not incorporate these poorly understood mechanisms. We acknowledge that this assumption might be another shortcoming of the computational model. Mechanisms associated with inhibition of MM cells on bone formation will be considered in future models.

**Figure 2:**
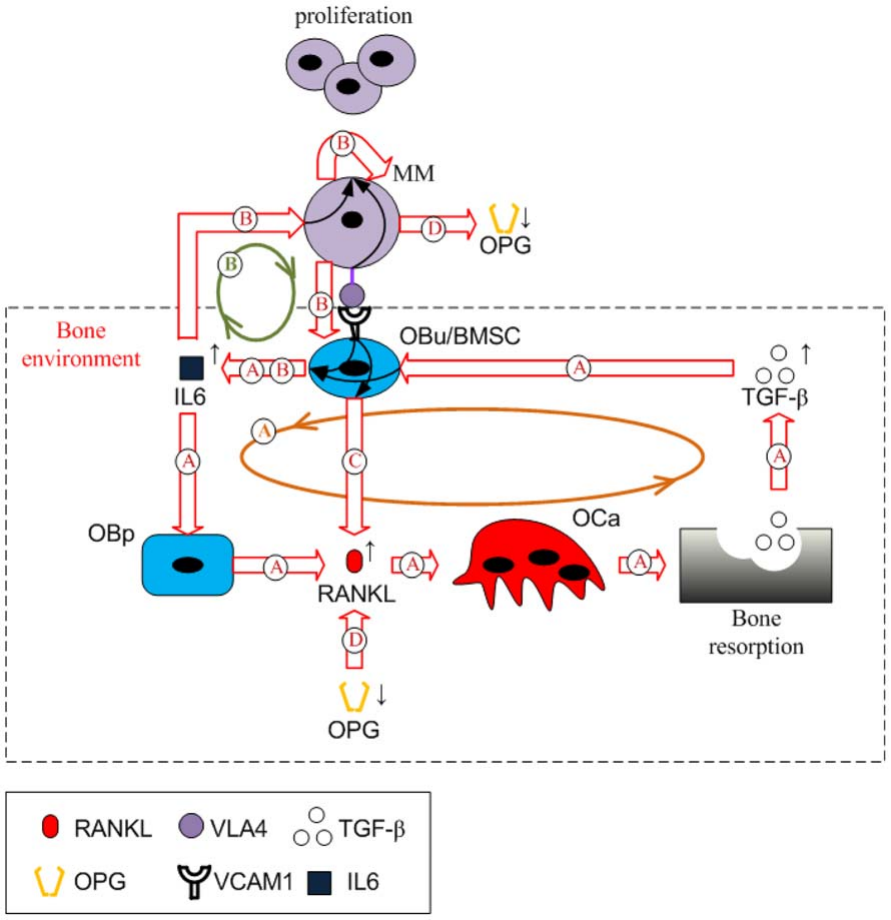
Schematic of the positive feedback cycles in the MM-bone model. The positive feedback loop A forms the first cycle within the bone microenvironment, which is enhanced by the increased IL-6 concentrations due to MM-BMSC adhesion. IL-6 secreted by BMSC stimulates elevated RANKL expression on the surface of osteoblast precursors and further increased active osteoclasts, leading to bone resorption and TGF-β released from bone resorption. Released TGF-β, in turn, stimulates more IL-6 secretion by BMSC. The positive feedback loop B forms the second cycle. Simultaneous stimulation of MM-BMSC adhesion and TGF-β induces substantial IL-6 secretion by BMSC, which (together with MM-BMSC adhesion) causes MM-cell proliferation and further enhanced MM-BMSC adhesion. The first and the second cycle interact with each other by enhancing IL-6 production. Two regulations, MM-BMSC adhesion stimulating RANKL expression on the surface of BMSC and MM-cell degrading OPG, enhance the positive feedback cycles of MM-bone interactions through increasing IL-6 concentrations.

Taken together, all the regulation mechanisms in this model are found to form two positive feedback cycles. As Figure 2 shows, IL-6 secreted by BMSCs induces increased RANKL expression on osteoblast precursors, leading to bone resorption. TGF-β released during bone resorption, in turn, stimulates the secretion of IL-6 by BMSCs. This ‘positive feedback loop’ forms a positive feedback cycle within the bone microenvironment (identified as cycle A in Figure 2), which is enhanced by the increased IL-6 concentrations due to MM-BMSC adhesion stimulation. This cycle does not normally have a significant effect on bone resorption, because TGF-β acting alone only stimulates a small increase in IL-6 secretion by BMSCs in the context of normal bone physiology. However, in the presence of MM cells and with the simultaneous stimulation of TGF-β and MM-BMSC adhesion, a substantial increase in IL-6 secretion by BMSCs occurs. The elevated IL-6 concentrations now contribute to the positive feedback cycle, producing significant impacts on bone resorption.

On the other hand, IL-6 and MM-BMSC adhesion stimulate MM-cell proliferation, which in turn promotes IL-6 production by BMSC and enhances MM-BMSC adhesion. This leads to another ‘positive feedback loop’, so forming a second cycle between MM cells and the bone microenvironment (identified as cycle B in Figure 2). Due to the dual roles played by IL-6 in these positive feedback cycles (both an osteoclastogenesis factor and a stimulatory factor of MM-cell proliferation), the first and the second cycles interact with each other, enhancing IL-6 production. This induces a positive feedback cycle between MM cells and the bone microenvironment triggered by either TGF-β or MM-BMSC adhesion. Two additional signaling pathways, MM-BMSC adhesion stimulating RANKL expression on the surface of BMSCs (identified as pathway C in Figure 2), and MM-cells degrading OPG (identified as pathway D in Figure 2), also serve to enhance the positive feedback cycles between MM cells and the bone microenvironment by increasing the RANKL/OPG ratio.

### 2.2 Cellular response to simultaneous stimulation by two ligands

In the bone remodeling model of Pivonka et al. [1], cell-cell regulatory communication is represented by chemical mass-action equations, while cellular process are represented by transfer functions, usually Hill functions of the form: 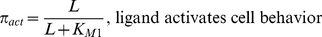
(1)

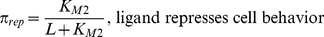
(2)where *π_act_* and *π_rep_* represent the ‘activator’ or ‘repressor’ function respectively; L is the ligand concentration; and *K_M_*_1_ and *K_M2_* represent the half-maximal concentrations respectively, which are the ligand concentrations inducing a half-maximal cell response.

In the proposed MM-bone model, three cases of cellular processes are simultaneously controlled by two ligands (rather than by a single ligand): (i) RANKL production by osteoblast precursors is co-regulated by PTH and IL-6, (ii) MM-cell proliferation is co-regulated by MM-BMSC adhesion and IL-6; and (iii) IL-6 production by BMSC is co-regulated by TGF-β and MM-BMSC adhesion. Accordingly, equations (2) and (3) (used in the Pivonka et al bone model [1]) need to be extended to appropriately model the cellular process in response to stimulation by two ligands (*L_1_* and *L_2_*).

The feature of simultaneous stimulation by two ligands is that there may exist intracellular interactions between the two separate ligand signaling pathways, which induce nonlinear cellular outputs. For our current needs, the intracellular interaction may induce an ‘enhanced’ cellular response (that is, a response that is greater than the cellular response to one ligand stimulation alone, but lower than the sum of cellular responses to each ligand stimulation acting separately), or a ‘synergistic’ cellular response (that is, a response that is greater than the sum of cellular responses to each ligand stimulation acting separately). For the intermediate case, the intracellular interaction may induce an ‘additive’ cellular response (that is, a response that is exactly equal to the sum of cellular responses to each ligand stimulation acting separately). The biological evidence indicates that RANKL production by osteoblast precursors is ‘enhanced’ under co-regulation by PTH and IL-6 [27], and that MM-cell proliferation is also ‘enhanced’ under co-regulation by MM-BMSC adhesion and IL-6 [23]; while IL-6 production by BMSC is ‘synergistic’ under co-regulation by TGF-β and MM-BMSC adhesion because the ratio of IL-6 production by two ligands stimulation compared to the sum of each ligand separately is between 1.45-fold and 2-fold [35].

Given the above description of the observed behaviors, mathematically, we define a response function 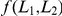 which meets the following constraints:

- There is at least a nonlinear term in the definition of the transfer function to reflect non-linear intracellular interactions between the two inputs.
- For the ‘enhanced’ response, the response function f is greater than either *π_1_* and *π_2_* but lower than the sum of *π_1_* and *π_2_* (i.e. *f* > *π_1_* and *f* > *π_2_* but *f* < *π_1_*+*π_2_*), while for the synergistic response, the response function *f* > *π_1_*+ *π_2_*.

The following function meets the above constraints (and has been successfully applied in modeling gene regulatory motifs [51] and synergistic effects of two inhibitors respectively [52], and so is a candidate response function to model cellular responses when there is stimulation by two ligands:
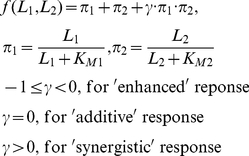
(3)where *π_1_* and *π_2_* represents the ‘activator’ function for each ligand, *L_1_* or *L_2_* (although we take the ‘activator’ function for example in here it should be noted that *π_1_* and *π_2_* can also represent the ‘repressor’ function for each ligand *L_1_* or *L_2_*), *L_1_* and *L_2_* are the ligand concentration respectively, and *K_M1_* and *K_M2_* are the half-maximal concentrations. *γ* is a parameter that may be calibrated to reflect the enhanced, the additive, or the synergistic effects that are observed experimentally.

**Figure 3:**
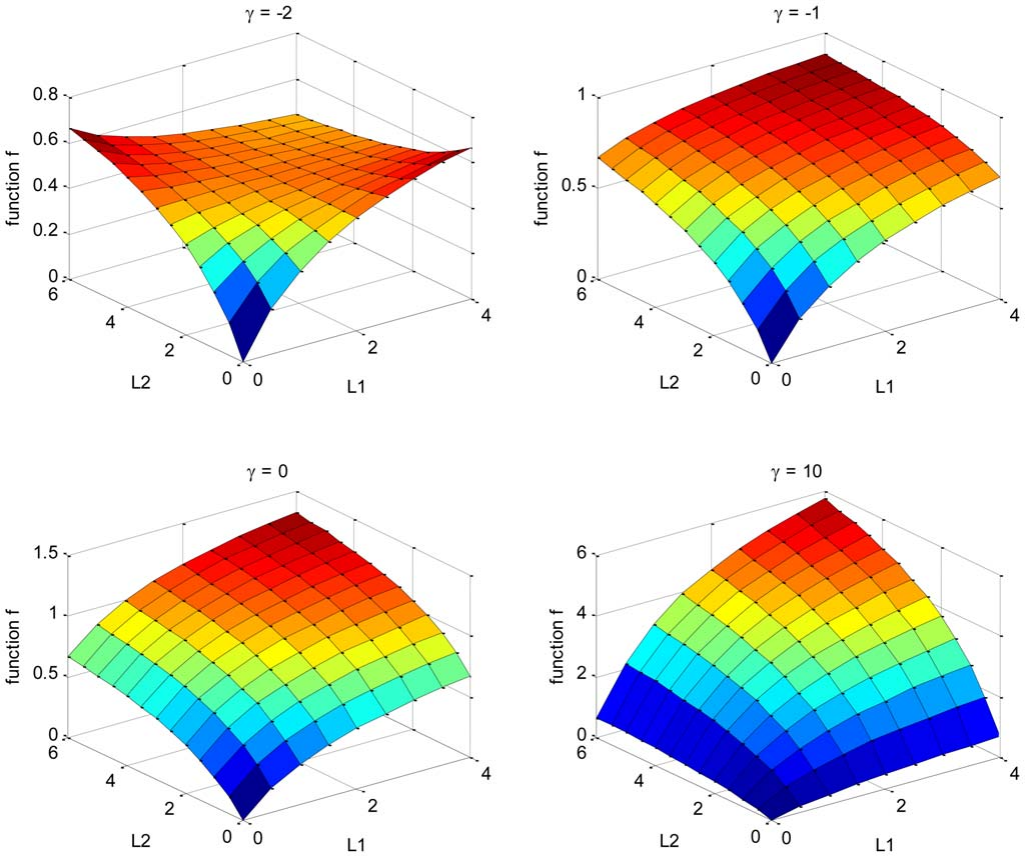
Functional dependence of f on *L_1_* and *L_2_* concentrations (*γ* = -2, -1, 0, and 10 respectively, *K_M1_* = 2 and *K_M2_* = 3).

Figure 3 illustrates the functional dependence of f on *L_1_* and *L_2_* concentrations. To meet the functional requirements described above, *γ* has to be greater than or equal to -1. For *γ* > -1, the function f increases with increase in *L_1_* and *L_2_* concentration, and the rate of change of f to an increase in ligand is more rapid with an increasing *γ* value. We choose *γ* to be -1 for the enhanced two-ligand interactions for simplicity, and *γ* is calibrated to be about 25 for the synergistic response. As demonstrated below, simulations using these parameter values appear to be in good agreement with observations for IL-6 and RANKL in normal bone physiology, and as demonstrated later, there is also good agreement at the various stages of MM disease.

## Results

### 3.1 Bone model in the absence of MM cells

In the absence of MM cells, several behaviors of normal bone physiology have been shown to be captured by the bone remodeling model of Pivonka et al. [1], [18]. However, to include the interactions between MM cells and bone cells adequately, this bone remodeling model needs to be extended to incorporate the mechanisms of TGF-β-stimulated IL-6 production by BMSC and IL-6-stimulated RANKL expression on the surface of osteoblast precursors. The newly introduced IL-6 should not significantly change the original behaviors between osteoclast and osteoblast functions as captured in the model of Pivonka et al. [1], [18] under normal bone conditions, as this has been extensively validated. Consequently, the IL-6 related parameters need to be carefully estimated, allowing that IL-6 produces insignificant impacts on the normal bone physiology, but does produce significant effects in the MM disease state.

In this next section, we present in detail how IL-6-related mechanisms are incorporated into the previous Pivonka et al. [1] model and demonstrate that our extended (MM-free) bone model correctly retains the osteoclast and osteoblast behaviors as occurs in the original model of Pivonka et al. [1], [18]. The calibration of the new extended bone model is made to quantitatively reflect the known minor IL-6 role(s) in normal bone physiology.

#### 3.1.1 Formulation of governing equations

According to the bone model of Pivonka et al. [1] the dynamic equations of describing bone cell populations are as follows:
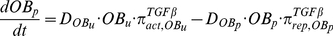
(4)

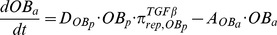
(5)

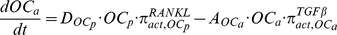
(6)where, OB_u_, OB_p_, OB_a_, OC_p_ and OC_a_ represent uncommitted osteoblasts, osteoblast precursors, active osteoblasts, osteoclast precursors and active osteoclasts respectively. *D_OBu_*, *D_OBp_* and *D_OCp_* represent the differentiation of OB_u_, OB_p_ and OC_p_ respectively. *A_OBa_* and *A_OCa_* represent the apoptosis of OB_a_ and OC_a_ respectively. 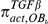, 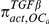 and 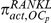 represent ‘activator’ functions while 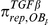 represents a ‘repressor’ function. These ‘activator’ and ‘repressor’ functions are defined by the Eq.(1) and (2) respectively and are the same as those in the model of Pivonka et al. [1].

Changes over time in the active osteoblast and osteoclast populations relative to each other, result in changes in bone volume, which is a ‘system output’ of the bone model. The calculation of bone volume is the same to that in Pivonka et al. [18]:
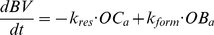
(7)where *BV* represents normalized bone volume, *k_res_* and *k_form_* represents relative rate of bone resorption and bone formation respectively with the unit of 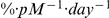.

In the extended bone model IL-6 production by BMSC/OB_u_s is stimulated by TGF-β. The dynamic equation describing IL-6 concentration is as follows:
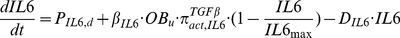
(8)


The molecular concentration changes and molecular reactions occur much faster than cellular changes, and as a result of this separation of time scales, it is appropriate to assume that molecules and receptors are always at equilibrium. The quasi-steady state concentration of IL-6 is solved from Eq.(8) as follows:
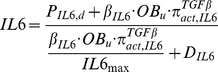
(9)

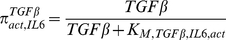
(10)where, *P_IL6,d_* is the external production rate of IL-6 with the unit of 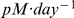. *β_IL6_* is the endogenous production of IL-6 by OB_u_ with the unit of 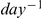. *IL6_max_* is the maximum concentration of IL-6. *D_IL6_* is the degradation of IL-6. 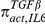 is the ‘activator’ function and *K_M,TGFβ,IL6,act_* is the half-maximal concentration of TGF-β promoting the production of IL-6.

RANKL production is controlled by both PTH and IL-6 in the extended bone model, and so the calculation of RANKL concentration at the steady state is updated from the Pivonka et al. model [1] as follows:
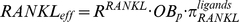
(11)

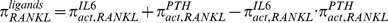
(12)

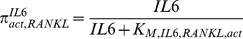
(13)

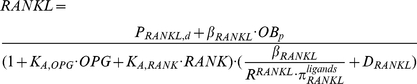
(14)where *RANKL_eff_* represents the ‘effective carrying capacity’ on the surface of osteoblast precursors, which is the maximum concentration of RANKL. *K_A,OPG_* and *K_A,RANK_* are the association rate constant for RANKL binding to OPG and RANK respectively. *R^RANKL^* is the maximal number of RANKL that can be expressed on the surface of osteoblast precursors. *P_RANKL,d_* is the external production rate of RANKL with the unit of 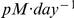. *β_RANKL_* is the endogenous production of RANKL by each OB_p_ cell with the unit of 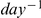. *D_RANKL_* is the degradation of RANKL. 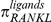 is the enhanced (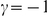) ‘activator’ function in response to simultaneous PTH and IL-6 stimulation (see discussion for Eq.(3)). 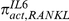 and 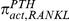 are the single ‘activator’ functions in response to PTH and IL-6 stimulation. *K_M,IL6,RANKL,act_* is the half-maximal concentration of IL-6 on promoting the production of RANKL.

The calculations of concentrations of TGF-β, PTH and OPG are the same as described in Pivonka et al. [1] (see Supporting Information S1).

#### 3.1.2 Perturbations on bone model

Before perturbations are performed to test the behavior of the extended bone model, this model has to reach the steady state representing normal bone physiology. To do this, the density of bone cells at steady state are re-estimated here based on reported values for adults available in the literature (see Table 2), and so are different to the bone-cell densities used by Pivonka et al. [1], [18]. The parameter values of the extended bone model are carefully estimated so that re-estimated bone-cell densities (see Table 2) are obtained when solved using the routine ‘fsolve’ in the Matlab (see parameter estimates in Table 3).

**Table 2:** The initial values of densities of bone cells and MM cells in the MM-bone model.

Variables	Values	Unit	References or estimation
OB_u_/BMSC^1^	3.27×10^−6^	pM	[21]; [76];
OB_p_^2^	7.67×10^−4^	pM	estimated;
OB_a_^3^	6.39×10^−4^	pM	[77]; [78];
OC_p_^4^	1.28×10^−3^	pM	[8];
OC_a_^5^	1.07×10^−4^	pM	[77]; [78];
MM^6^	3.26×10^−1^	pM	[79]; [57];

Note 1: BMSC is 1/2.5×10^5^ of total bone marrow cells in adults [21]; the estimated number of total cells in leg bone marrow is 4.4×10^11^ (#) (http://bloodguys.com/blood-education); the volume of bone marrow in leg is 8.6% of total bone marrow volume [76]; By assuming that cells in bone marrow are evenly distributed in different bone types, the number of BMSC in adults is 2.05×10^7^ (#) ( = 4.4×10^11^/8.6%/2.5×10^5^). Given that MM is generally occurred in the elder people and BMSC percentage in bone marrow decreases to 1/2×10^6^ in elder people aged 80 [21], the estimated BMSC number is corrected to ¼ of the number in adults, namely 5.12×10^6^ (#).

Note 2: It is assumed to be 1.2-fold greater than the number of active osteoblasts (OB_a_).

Note 3: There are 1∼2×10^6^ BMU [77] in the total body while there are about 10^2^∼10^3^ active osteoblasts (OB_a_) per BMU [78]. Hence, we estimate OB_a_ numbers as 1×10^9^ (#).

Note 4: Active osteoclasts (OC_a_) includes 9 nuclei [8] because they are fused by osteoclast precursors (OC_p_) differentiated cells. By assuming that OC_p_ is 12-fold of OC_a_, The estimated OC_p_ number is 2×10^9^ (#).

Note 5: There are about 10∼10^2^ active osteoclasts (OC_a_) per BMU [78]. Hence, we estimate OC_a_ number as 1.67×10^8^ (#).

Note 6: Synthesis rate of M-protein by MM cells is 0.5∼1.2×10^−11^ g/day/MM cell; the half-life of M-protein is 11.6∼17 days [79]; the volume of total blood in the adult is 5L; the diagnosis of MM is required the concentration of M-protein is greater than 30 g/L [57]. As a result, the estimated MM cell number at the diagnosis is 5.1×10^11^ (#) ( = 30×5× (log(2)/17)/1.2×10^−11^).

Note 7: All the estimated cell numbers (#) are based on the total human body. They are converted into density (pM) by divided by Avogadro number (6.02×10^23^ #/mol) and the volume of total bone marrow, which is estimated 2.6L because the estimated mass of total bone marrow is 2.6 kg (http://en.wikipedia.org/wiki/Bone_marrow) and the marrow density is assumed to be close to water.

**Table 3:** The parameter values in the (MM-free) normal bone model.

Parameters	Values	Unit	References or estimation
D_OBu_	2.94e+2	/day	estimated;
D_OBp_	3.57e-1	/day	estimated;
A_OBa_	3e-1	/day	[1];
D_OCp_	2e-1	/day	estimated;
A_OCa_	1.2	/day	[1];
K_M,TGFβ,act_	4.28e-4	pM	[1];
K_M,TGFβ,rep_	2.49e-4	pM	[1];
K_M,PTH,act_	2.09e+2	pM	[1];
K_M,PTH,rep_	2.21e-1	pM	[1];
K_M,TGFβ,IL6,act_	2.9e-3	pM	estimated;
K_M,IL6,RANKL,act_	8.8	pM	estimated;
K_M,RANKL,act_	4.79e+1	pM	[1];
α	1	pM/%	[1];
D_TGFβ_	2e+2	/day	[80];
β_PTH_	9.74e+2	pM/day	[81];
D_PTH_	3.84e+2	/day	[81];
β_IL6_	1.2e+7	/day	[36]; [24];
D_IL6_	4.99e+1	/day	[82];
IL6_max_^1^	8.04e-1	pM	[75];
β_OPG_	3.42e+6	/day	estimated;
D_OPG_	4.16	/day	[83];
OPG_max_^2^	7.98e+2	pM	[69];
β_RANKL_	3.37e+5	/day	estimated;
D_RANKL_	4.16	/day	[83];
R^RANKL^	3e+6	-	[1];
RANK	1.28e+1	pM	[1];
K_A,OPG_	5.68e-2	/pM	[84];
K_A,RANK_	7.19e-2	/pM	[84];
k_res_	2e+2	%/(pM*day)	[18];
k_form_	3.34e+1	%/(pM*day)	estimated;

Note 1: It is assumed to be 30-fold greater than IL-6 concentration at steady state.

Note 2: It is assumed to be 20-fold greater than OPG concentration at steady state.

After this calibration, various perturbations of this model are performed from steady state (by adding or removing cells or signaling molecules) and the effects of the perturbations are compared with the responses obtained in Pivonka et al. [1], [18]. This allows us to clarify whether this extended bone model is able to keep the original changes in density of bone cells and keep the original changes in bone volume (as reported in the Pivonka et al. model [1], [18]). Perturbed cells or signaling molecules are expected to quickly reach a new steady state and to quickly recover to the original steady state when perturbations are removed, just as they did in the Pivonka et al. model [1], [18]. In the series of tests, all the perturbations are added at day 20 and end at day 80 (after the extended bone model has reached a new steady state). The dynamic simulations are implemented using the routine ‘ode15s’ in the Matlab.

To evaluate whether IL-6 related parameters are well estimated (to quantitatively reflect the IL-6 roles in this extended bone model), ratios of 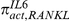 and 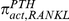 to 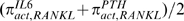 is calculated respectively. If IL-6 and PTH equally contribute to RANKL production, both ratios are equal to 1. If IL-6 dominantly contributes to RANKL production, the ratio 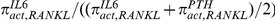 is greater than 1 while the ratio 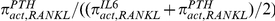 is lower than 1. On the contrary, if PTH dominates the contribution to RANKL production, the ratio 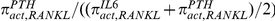 is greater than 1, while the ratio 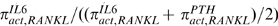 is lower than 1. Based on the ratios, the quantitative roles of IL-6 in RANKL production and impacts of IL-6 on the bone microenvironment may be assessed.

The outcomes of the perturbation test series are summarized in Table 4. In all perturbation tests, bone cells or signaling molecules quickly reach a new steady state following perturbations and quickly recover to the original steady state after the perturbations are removed, which is consistent with the response behavior in the Pivonka et al. model [1], [18]. In terms of changes of bone volume, the modified model fully aligns with the Pivonka et al. model [1], [18] for all test perturbations. It is worth noting that bone volume depends on the history of the bone cell populations and so bone volume reaches a new value after removing perturbations rather than returning to the original value. All these observations demonstrate that the new extended bone model incorporating the new IL-6 related control functions behaves essentially identically with the Pivonka et al. model [1], [18].

**Table 4:** Outcomes of perturbations on the (MM-free) normal bone model.

Perturbations	Bone cells(OB_p_, OB_a_, OC_a_)	Bone volume	Molecules(OPG, RANKL, IL-6)
OB_p_ ↑Adding 8e-5 pM/day	↑	↑, to new value;above normal;	↑
OB_a_ ↑Adding 6e-5 pM/day	OB_a_ ↑ but OB_p_ and OC_a_ ↓	↑, to new value;above normal;	OPG ↑ but RANKL and IL-6 ↓
OC_a_ ↑Adding 1e-5 pM/day	↑	↓ then ↑ tonew value;below normal;	↑
OB_p_ ↓Removing 3e-5 pM/day	↓	↓, to new value;below normal;	↓
OB_a_ ↓Removing 2e-5 pM/day	OB_a_ ↓ but OB_p_ and OC_a_ ↑	↓, to new value;below normal;	OPG ↓ but RANKL and IL-6 ↑
OC_a_ ↓Removing 3e-6 pM/day	↓	↑ then ↓ to new value;above normal;	↓
PTH ↑Adding 1e+3 pM/day	↑	↓ then ↑ tonew value;below normal;	RANKL and IL-6 ↑ but OPG ↓
OPG ↑Adding 2e+2 pM/day	↓	↑ then ↓ to new value;above normal;	OPG ↑ but RANKL and IL-6 ↓
RANKL ↑Adding 3e+2 pM/day	↑	↓ then ↑ tonew value;below normal;	↑
IL-6 ↑Adding 10 pM/day	↑	↓ then ↑ tonew value;below normal;	↑
OPG ↓Removing 5e+1 pM/day	↑	↓ then ↑ tonew value;below normal;	OPG ↓ but RANKL and IL-6 ↑

Note 1: All perturbation responses in (MM-free) bone model (except IL-6 perturbation response) are qualitatively consistent with those of Pivonka et al. model [1], [18].

Note 2: This table summarizes transient changes of state variables (i.e. densities of bone cells and molecule concentrations) after adding perturbations, while it summarizes transient changes in bone volume after adding perturbations together with the new state of bone volume reached after removing perturbations.

**Figure 4:**
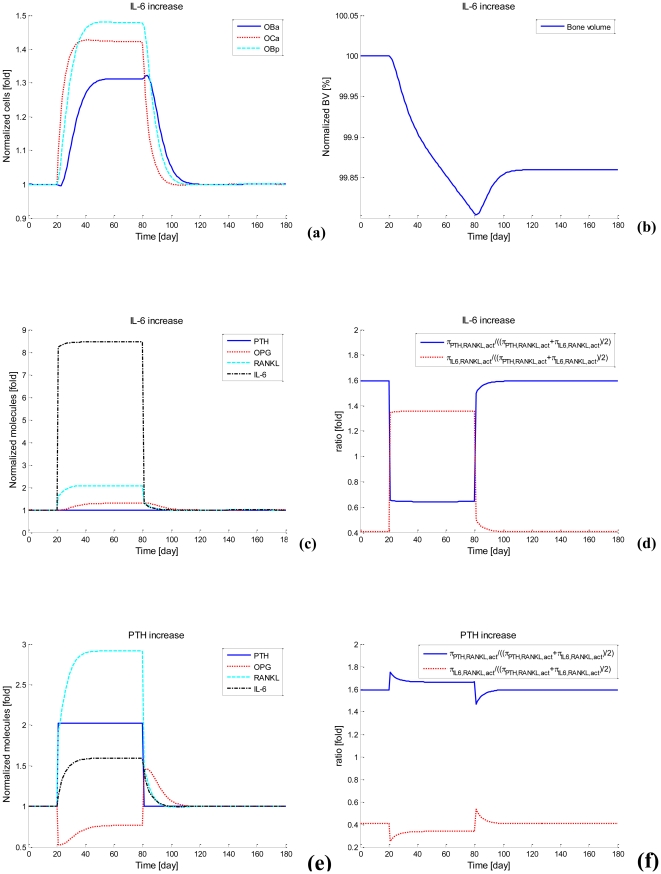
Perturbations of the (MM-free) normal bone model. (a) Bone cells after adding IL-6. (b) Bone volume after adding IL-6. (c) Molecules after adding IL-6. (d) Ratios of 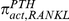 and 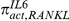 to 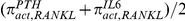 respectively after adding IL-6. (e) Molecules after adding PTH. (f) Ratios of 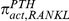 and 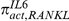 to 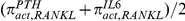 respectively after adding PTH.

At the steady state, the ratio 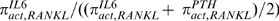 and 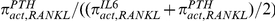 are found to be 0.4 and 1.6 respectively, indicating the IL-6 contribution to RANKL production is not dominant in the case of normal bone physiology. This has been achieved through appropriate model calibration. In all perturbation cases (except for adding IL-6 perturbation), the IL-6 concentration is not significantly changed and its contribution to RANKL production is not significantly increased under normal bone conditions. For example, when PTH is added at a rate of 1000 pM/day, concentration of PTH, IL-6 and RANKL increase 2-fold, 1.6-fold and 3-fold respectively (Figure 4e), while the ratio 
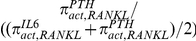
 increases with a simultaneous decreased ratio 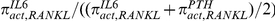 (Figure 4f). This indicates that TGF-β activation does not induce a substantial increase in IL-6 production in the extended bone model. In other words, under normal bone conditions, IL-6 does not make a dominant contribution to RANKL production.

When IL-6 is injected at a rate of 10 pM/day, IL-6 concentration increases eight-fold while RANKL concentration increases two-fold (Figure 4c), leading to increase in osteoclast bone cell densities and a decrease in bone volume (see Figure 4a-b). In addition, the ratio 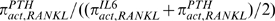 increases from 0.4 to 1.4 while the ratio 
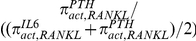
 decreases from 1.6 to 0.6 (Figure 4d), indicating the relative contribution of IL-6 to RANKL production is dominant. The IL-6 perturbation analysis demonstrates that substantial increase in IL-6 is able to produce significant impacts on the RANKL production and so has a strong influence on the bone microenvironment.

These results show that the new extended bone model has been carefully calibrated such that under normal conditions, IL-6 only does not play a dominant role in bone physiology because of its moderate regulation of RANKL production. However, if some mechanism or mechanisms trigger a significant increase in IL-6 in the bone microenvironment, the IL-6 contribution to RANKL production can become dominant, inducing significant increase in RANKL concentration (and consequent increase in densities of osteoclastic bone cells and bone resorption). Therefore, the Pivonka et al. model [1] has been suitably extended to take into account the actions of IL-6 in normal bone physiology in preparation for modeling MM disease states.

### 3.2 MM-bone model

#### 3.2.1 Formulation of governing equations

In the presence of MM cells in the bone environment, MM cells proliferate due to stimulation by IL-6, MM-BMSC adhesion and other events, and die due to T-cell interactions and other actions. The population of MM cells is seen to increase in an S-shape fashion in a few biological experiments [53], [54]. In terms of mathematical modeling, a logistic function is widely used to model the S-shape increase in tumor cells [55]. Consequently the population growth of MM cells is modeled here using a logistic function. By assuming the apoptosis of MM cells is a first-order process, with the rate of the apoptosis proportional to the density of MM cells [56], the dynamics of MM-cell population satisfies the following equations:
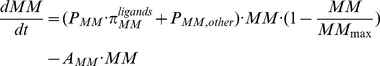
(15)

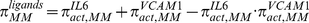
(16)

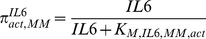
(17)

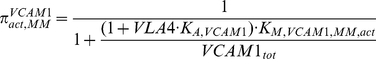
(18)where, *P_MM_* is the proliferation of MM cells controlled by IL-6 and MM-BMSC adhesion. *P_MM,other_* is the proliferation of MM cells controlled by other events (e.g., IGF-1 rather than arising from IL-6 and MM-BMSC adhesion). *A_MM_* is the apoptosis of MM cells. 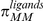 is the enhanced (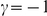) ‘activator’ function in response to simultaneous MM-BMSC adhesion and IL-6 stimulation (as defined by the Eq.(3)). 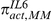 and 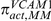 are the ‘activator’ functions in response to IL-6 and MM-BMSC adhesion stimulation separately. *K_M,VCAM1,MM,act_* and *K_M,IL6,MM,act_* are the half-maximal concentration of VLA-4 and IL-6 on facilitating MM-cell proliferation. *K_A,VCAM1_* is the association rate constant for VLA-4 binding to VCAM-1. *VCAM1_tot_* is the total concentration of VCAM-1. The details to derive Eq.(18) are described in Supporting Information S1.

The membrane bound VLA-4 concentrations at steady-state are calculated in much the same way as RANKL concentrations (see equation (31)-(36) in Pivonka et al. [1]), via:
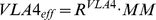
(19)

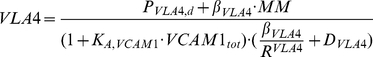
(20)where *VLA4_eff_* represents the ‘effective carrying capacity’ on the surface of MM cells, which sets the maximum concentration of VLA-4. *P_VLA4,d_* is the external production rate of VLA-4 with the unit of 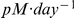. *K_A,VCAM1_* is the association rate constant for VLA-4 binding to VCAM-1. *R^VLA4^* is the maximal number of VLA-4 that can be expressed on the surface of MM cells. *β_VLA4_* is the production of VLA-4 by MM with the unit of 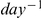. *D_VLA4_* is the degradation of VLA-4. *VCAM1_tot_* is the total concentration of VCAM-1.

In the presence of MM cells, IL-6 production is stimulated not only by TGF-β but also by MM-BMSC adhesion. To account for this, the calculation of IL-6 concentration in updated form (from Eq.(9)) is as follows: 
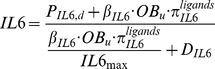
(21)

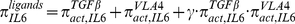
(22)

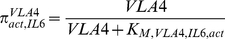
(23)where, 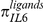 is a synergistic ‘activator’ function in response to VLA-4 and simultaneous TGF-β stimulation (its calculation is defined by Eq.(3)). 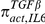 and 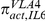 are ‘activator’ functions in response to TGF-β and MM-BMSC adhesion stimulation. *K_M,VLA4,IL6,act_* and *K_M,TGFβ,IL6,act_* are the half-maximal concentrations of VLA-4 and TGF-β respectively, when promoting the production of IL-6.

By assuming the internalization and degradation of OPG by MM cells is proportional to OPG concentrations and the density of MM cells, the calculations of OPG concentration is updated from [1] as follows:
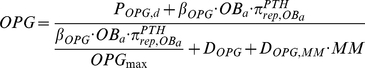
(24)where, *P_OPG,d_* is the external production rate of OPG with the unit of 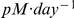. *β_OPG_* is the endogenous production of OPG by active osteoblasts with the unit of 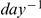. *OPG_max_* is the maximal concentration of OPG produced by active osteoblasts. *D_OPG_* is the degradation of OPG. *D_OPG,MM_* is the degradation of OPG by MM cells. 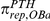 is the ‘repressor’ function, which is the same as that in [1].

In the presence of MM cells, RANKL is produced not only by the osteoclast precursors (OB_p_), but also by the uncommitted osteoblasts (OB_u_) as a result of MM-BMSC adhesion. Our estimations suggest that the number of OB_u_ cells is two orders of magnitude lower than the number of OB_p_ cells, that is, OB_u_ << OB_p_ (see Table 2). Consequently, RANKL production by OB_u_ cells is thought to contribute little to the total RANKL concentrations in the context of MM disease, and so this contribution may be neglected without significantly influencing MM disease progression. For this reason, the calculation for the RANKL concentrations in the presence of MM cells is the same as Eq.(14).

#### 3.2.2 Bifurcation

Eq.(15) represents the balance between a source term (due to the proliferation of MM cells) and a sink term (due to the apoptosis of MM cells). The density of MM cells may increase or decrease depending on parameter values. Clearly, if the source term is greater than the sink term, MM-cell density increases, while if source term is smaller than the sink term, MM-cell density decreases. A dynamic increase in MM-cell density may suddenly revert to the dynamic decrease in MM-cell density at a critical condition, and vice versa. In other words, a bifurcation might occur in the MM cell population equation (Eq.(15)), which is determined by the critical condition.



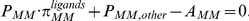



If 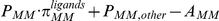 >0, MM-cell density increases;

If 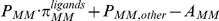  = 0, MM-cell density remains constant;

If 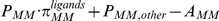 <0, MM-cell density decreases.

In order to ensure an increase in MM-cell density during simulations of the MM-bone model, the first condition has to be met. Because *P_MM,other_* is assumed to be very small in the model, the critical condition is mainly determined by the parameters *P_MM_*, *A_MM_* and 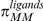. While *P_MM_* and *A_MM_* are independent of time, 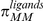 is time dependent because it is a function of the concentrations of IL-6 and VLA-4, which are time dependent. At the beginning of simulation, the concentrations of IL-6 and VLA-4 can be calculated from the steady state of normal bone model and the initial density of MM cells respectively; and so the value of 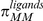 is estimated at the beginning of simulation. *A_MM_* is estimated as 2×10^−3^/day according to the literature; consequently, *P_MM_* must be greater than 2.87×10^−2^/day to meet the first condition, ensuring MM cells increase from the beginning of simulation (note that MM cell density may subsequently decrease if *P_MM_* changes during the simulation).

#### 3.2.3 Simulations

In order to simulate the transient behavior of the MM-bone model following the introduction of MM cells, the initial state for MM cells needs to be estimated. MM disease progression is clinically divided into three phases according to criteria for classification recommended by IMWG [57]: (i) monoclonal gammopathy of undetermined significance (MGUS); (ii) so-called ‘smoldering MM’ (or asymptomatic MM); (iii) malignant MM (or symptomatic MM). Malignant MM is associated with bone lesions, while MGUS and smoldering MM does not exhibit bone lesions. Because our primary concern is MM-induced bone destruction, our simulations are focused on phase (iii)-malignant MM. Furthermore, malignant MM (phase (iii)) is further divided into three clinical stages (namely, stages I, II and III), based on staging system of Durie-Salmon (or some alternate combination of prognostic factors) [58], [59]. The median survival duration following diagnosis is currently in the range 50–55 months [60]. As a result, our simulations here start from the early part of stage I in phase (iii) of MM, and end at the end of stage III, phase (iii), with this transition occurring over a time period of about 60 months. For these circumstances, the initial density of MM cells is estimated as 0.326 pM (see Table 2).

The parameter values of the MM-bone model are given in Table 5. These parameters are estimated based on reported values in the literature, together with best-fitting model estimates from least-square optimization criteria (i.e. parameters *β_VLA4_*, *D_OPG,MM_* and *γ* are optimized using this criteria). It should be noted that although there are a lot of parameters, the values for most of them are not obtained by fitting procedures. About 80% of model parameters are pathophysiology-related and estimated based on experiments reported in the literature. The optimization criterion used in this paper is to minimize the errors between simulations and experiments or clinical observations, that is, to minimize the following objective function: 
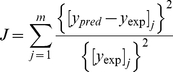
(25)


Where, the *y_pred_* and *y_exp_* are normalized simulated values and corresponding experimental or clinical values respectively, at the end of simulations. The *j* represents different variables (specifically, they refer to MM-cell density, IL-6 concentrations, RANKL concentrations and OPG concentrations). The values of *[y_pred_]_j_* are calculated using the routine ‘ode15s’ in the Matlab and the values of *[y_exp_]_j_* are estimated based on clinical observations (specifically, MM-cell density is estimated to increase 5-fold, IL-6 concentration to increase 10-fold, RANKL concentration to increase 4-fold and OPG concentration to decrease 0.7-fold, at the end of the simulations (see Table 6)). The optimization process is implemented using the routine ‘patternsearch’ in Matlab.

**Table 5:** The parameter values in the MM-bone model.

Parameters	Values	Unit	References or estimation
P_MM_	5.5e-2	/day	estimated;
P_MM,other_	2e-4	/day	estimated;
A_MM_	2e-3	/day	[85];
MM_max_	1.98	pM	[79];
K_M,VCAM1,MM,act_	8.07e-2	/pM	estimated;
K_M,VLA4,IL6,act_	3.36e+5	/pM	estimated;
K_M,IL6,MM,act_	1.76	pM	estimated;
β_VLA4_	2.74e+6	/day	calibrated;
D_VLA4_	2	/day	estimated;
R^VLA4^	5.6e+4	-	[86];
VCAM1_tot_	1.92	pM	[86];
K_A,VCAM1_	8.3e-2	/pM	[87];
D_OPG,MM_	4.11	/(pM*day)	calibrated;
γ	-1 (enhanced response) or2.47e+1 (synergistic response)	-	estimated for enhanced response while calibrated for synergistic response;

Note 1: The external dosing rate *P_VLA4,d_*, *P_IL6,d_*, *P_OPG,d_* and *P_RANKL,d_* are all set to zero.

**Table 6:** Comparisons of the MM-bone model outcomes under the condition of *P_MM_* = 0.055/day with experimental observations.

	Stage I/II		Stage III	
	experiments	simulations	experiments	simulations
RANKL	1.62-fold [70]	1.75-fold	2.65-fold [69];2.26-fold [70];13.5-fold [71];15.67-fold [72];	4.35-fold
IL-6	2.6-fold/4.22-fold [75]	3.55-fold	9.79-fold [75]	10-fold
OPG	↓ [69];↑ [88] ^1^	↓	0.71-fold [73];0.73-fold [38];0.82-fold [74];0.59-fold [69]	0.69-fold
OB_a_	↑ [67], [68], [89]	↑	↑ [67], [68], [89]	↑
OC_a_	↑ [67], [68], [89]	↑	↑ [67], [68], [89]	↑
Bone turnover	↑ [67], [68], [89]	↑	↑ [67], [68], [89]	↑
Bone volume	↓ [67]	↓	↓ [67]	↓
MM cells	3-fold [67]	↑	Up to 6-fold [67]	4.48-fold

Note 1: Clinically, it is observed that serum OPG concentrations decrease at the early stage of MM disease [69], while it is recently suggested that serum OPG concentrations increase compared with healthy controls [88]. The exact reasons to cause the different observations are still not known. Possibly, OPG is produced by various skeletal and extra-skeletal tissues [90], leading to serum OPG concentrations do not reflect its availability in the bone microenvironment [88].

Note 2: All the ratios of experiments are obtained by comparing with healthy controls, whereas all the ratios of simulations are obtained by comparing with steady state of the normal bone model.

**Figure 5:**
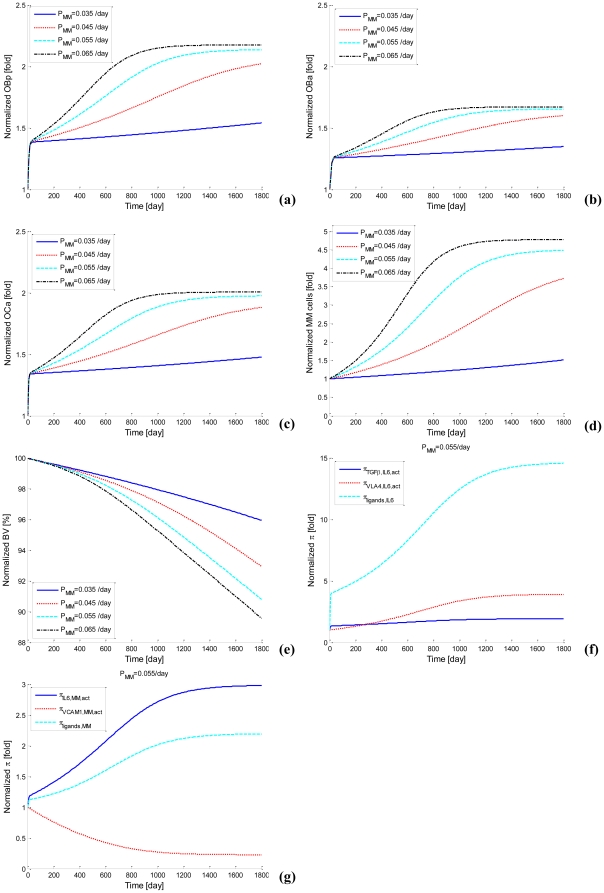
Simulations of the MM-bone model for various *P_MM_* Values. (a) OB_p_. (b) OB_a_. (c) OC_a_. (d) MM cells. (e) Bone volume. (f) The ‘activator’ function for IL-6 production (*P_MM_* = 0.055/day). (g) The ‘activator’ function for the proliferation of MM cells (*P_MM_* = 0.055/day).

With the calibrated parameter values (e.g., *P_MM_* equals 0.055/day), transient behavior of bone cells, MM cells and bone volume are simulated. The code has been checked in accordance with a comprehensive comparison of the simulation results against clinical data as reported in the literature (refer Section 3.2.5). Good agreement has been demonstrated. As cyan dash lines in Figure 5a-e show, MM cells and bone cells increase quickly and approach an upper limit after about 4 years, while bone volume continuously decreases. This curve represents a progressive MM disease process and approaches an upper limit at about the mean time for death that is observed clinically. It is worth noting that bone cells exhibit a sharp increase at the very beginning of simulations followed by an S-shape increase until the end state. Because we simulate the MM development in bone marrow by starting from stage I phase (iii) of MM (initial density of MM cells is 0.326 pM), this sharp increase is artificial and actually driven by the accumulated increase in bone cells during the period from the start of MM until the beginning of stage I phase (iii). After this sharp increase, the S-shape increase represents increased bone cells due to the MM disease over the period from stage I phase (iii) to stage III phase (iii).

Furthermore, as indicated by sensitivity analysis (see following section) the population growth of MM cells is most dependent on the proliferation of MM cells (the parameter *P_MM_*), and so the influence of *P_MM_* on the MM-cell population growth and MM-induced bone resorption are considered further. For different *P_MM_* values, different evolutions of MM are observed in the model (see Figure 5a-e). For example, when *P_MM_* equals 0.035/day, MM cells and bone cells slightly increase and bone volume loss remains small. This curve indicates that MM is only slightly progressive. When *P_MM_* equals 0.045/day, MM cells, bone cells and bone volume loss increases more. When *P_MM_* equals to 0.065/day, MM cells and bone cells increase more quickly and reach the upper limit by about 3 years, while reducing bone volume more significantly. This curve represents a more rapid progression of MM than the mean time observed clinically.

Figure 5f shows that the ‘activator’ function for IL-6 production, simultaneously stimulated by TGF-β and MM-BMSC adhesion, increases fourteen-fold at the end of simulation, while ‘activator’ function of IL-6 production stimulated by either TGF-β or MM-BMSC adhesion increase by only two-fold and four-fold respectively. The ratio of IL-6 production by two ligands stimulation compared to the sum of each ligand separately is over two-fold at the end of simulation, confirming the ‘synergistic’ effects of simultaneous TGF-β and MM-BMSC adhesion stimulation on the IL-6 production.

Clearly, as can be seen from Figure 5g, the ‘activator’ function of MM-cell proliferation stimulated by IL-6 increases three-fold at the end of simulation, while the ‘activator’ function of MM-cell proliferation simulated by MM-BMSC adhesion decreases to 20% of its original value However the ‘activator’ function of MM-cell proliferation simultaneously stimulated by IL-6 and MM-BMSC adhesion stimulation increase about two-fold at the end of simulations. This suggests that MM-cell proliferation mainly results from IL-6 stimulation as the MM disease process progresses, while MM-BMSC adhesion contributes to a lesser amount to MM-cell proliferation.

#### 3.2.3 Sensitivity analysis

In order to clarify the impacts of various parameters on MM disease progression, a sensitivity analysis is performed to identify critical parameters. The sensitivity is defined as the relative change of an output variable *v_i_* to the relative change in an input variable *p_j_* at the relevant time point, The sensitivity may be calculated as [61]:
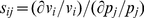
(26)


Given that the sensitivity estimate found from Eq.(26) is evaluated at a single time, while we are concerned with temporal changes of output variables over a period of 60 months, we extend this concept using the time integral 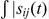
starting from the beginning of the simulation and finishing at the endpoint of the simulation. The greater the integral, the greater the time averaged sensitivity between a specific output variable and a specific input variable.

**Figure 6:**
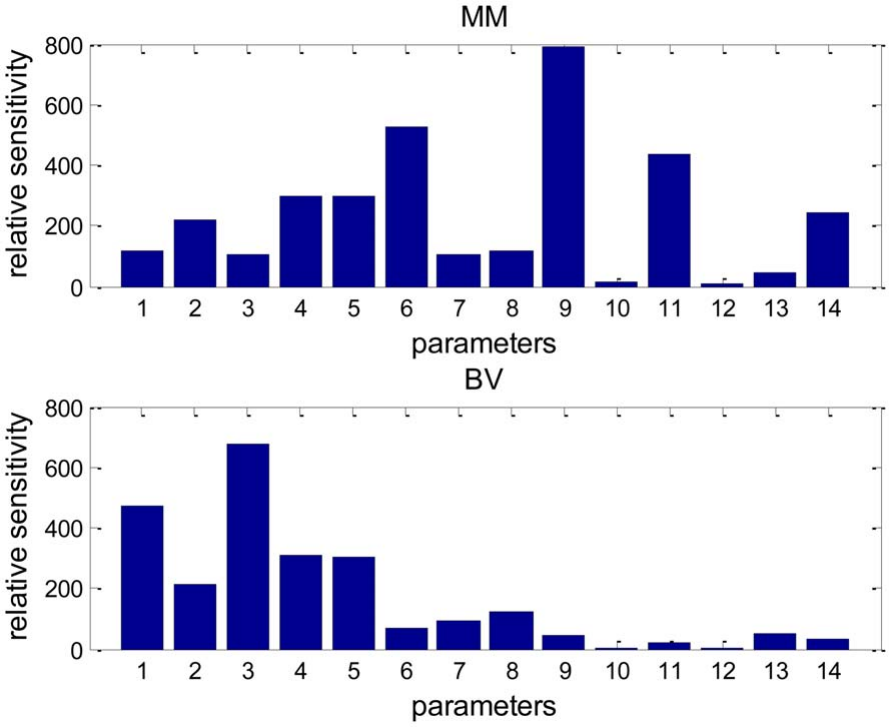
Outcomes of relative sensitivity analysis. 1-*D_OBu_*; 2-*D_OBp_*; 3-*A_OBa_*; 4-*D_OCp_*; 5-*A_OCa_*; 6-*β_IL6_*; 7-*β_OPG_*; 8-*β_RANKL_*; 9-*P_MM_*; 10-*P_MM,other_*; 11-*A_MM_*; 12-*β_VLA4_*; 13-*D_OPG,MM_*; 14-*γ*.

Figure 6 shows the outcomes of such a sensitivity analysis. It is apparent that the density of MM cells is most sensitive to *P_MM_*, *β_IL6_*, *A_MM_*, *D_OCp_* and *A_OCa_* while bone volume is most sensitive to *A_OBa_*, *D_OBu_*, *D_OCp_* and *A_OCa_*. Because parameters *P_MM_*, *β_IL6_* and *A_MM_* are directly associated with MM-cell density they are regarded as MM-related parameters. Likewise, parameters *A_OBa_*, *D_OBu_*, *D_OCp_* and *A_OCa_* are directly associated with bone-cell density and bone volume and so they are regarded as bone-related parameters (*β_IL6_* may also be regarded as bone-related parameter due to the dual roles of IL-6). More specifically, *A_OBa_*, and *D_OBu_* are osteoblast-related parameters whereas *D_OCp_* and *A_OCa_* are osteoclast-related parameters. Outcomes of the sensitivity analysis suggest that MM-cell density are most sensitive to MM-related group of parameters, and next most sensitive group are the osteoclast-related parameters, while bone volume is most sensitive to osteoblast-related group of parameters and next most sensitive to osteoclast-related parameters. Bone volume seems least sensitive to MM-related parameters while MM-cell density seems least sensitive to osteoblast-related parameters. These outcomes appear to be consistent with experimental or clinical observations. For example, anti-catabolic agents (e.g., an inhibitor of osteoclast activity or a specific inhibitor of RANKL) halt MM-induced bone resorption and result in inhibition of MM cell proliferation and survival [62], [63]. However, osteolytic lesions may still progress even if patients with MM respond to anti-MM therapy [64], [65].

#### 3.2.3 Detailed comparisons of model outcomes and clinical observations

The main goal of this paper is to clarify whether this proposed MM-bone model appropriately reflects clinical data on MM disease progression in bone marrow. The observed major clinical features of MM disease include: the increased bone resorption markers (i.e., N-terminal telopeptides of type I collagen, NTX) and bone formation marker (i.e., bone-specific alkaline phosphotase, bALP) [66], indicating an increase in the number of osteoclasts and osteoblasts as a result of increased RANKL and decreased OPG concentrations. The elevated osteoclast and osteoblast activities lead to increased bone turnover and reduced bone volume [66]. Malignant plasma cells (MM cells) in bone marrow secrete paraprotein (i.e., Bence-Jones proteins) and prognostic indicators of MM disease (e.g., IL-6), which increase as MM disease progresses [66]-[68].

Further, relative to normal healthy subjects, serum sRANKL (soluble RANKL) concentrations increase about one and a half fold in patients with stage I or stage II MM. Serum RANKL concentrations increase more variably later in the course of the disease. For example, relative to normal healthy subjects, serum RANKL concentrations are reported to increase 2.65-fold [69], 2.26-fold [70], 13.5-fold [71], and 15.67-fold [72] in patients with stage III MM. Relative to healthy controls, serum OPG concentrations have recently been reported to increase about one and half fold in patients with early stage MM [70], however in late stage MM, serum OPG concentrations have been reported to decrease 29% [73], 27% [38], 18% [74] and 41% [69]. Relative to healthy controls, serum IL-6 concentrations have been reported to increase 2.57-fold, 4.22-fold and 9.79-fold in MM patients at stages I, II and III respectively [75]. For patients with MM, measureable concentrations of IL-6 and sIL-6R are found in both marrow fluid and serum patients, and both fluids show similar increases in concentration [66]. The number of malignant plasma cells in bone marrow can account for up to 65% of the total number of cells in the bone marrow [67]. This is over six times higher than the clinical diagnosis criteria for MM with MM cells comprising 10% of the total number of cells. All these clinical observations are summarized in Table 6.

Most importantly, comparisons between the model outcomes and the above-mentioned clinical observations demonstrate that our simulation qualitatively and quantitatively agrees with these clinical observations. As summarized in Table 6, at the early stage I phase of MM, simulated RANKL and IL-6 concentration increase about 1.75-fold and 3.55-fold respectively, while clinical observations indicate that RANKL and IL-6 concentration increase about 1.62-fold and 2.6-fold respectively [70], [75]. For the MM example with *P_MM_*  =  0.055/day, at the endpoint of simulation (corresponding to later stage III phase of MM), simulated concentrations of OPG, RANKL and IL-6 and MM-cell density increase 0.69-fold, 4.35-fold, 10-fold and 4.48-fold respectively, while clinical observations suggest that concentrations of OPG, RANKL and IL-6 and MM-cell density increase approximately 0.7-fold [38], [73], 4-fold [69], [70], 10-fold [75] and 5-fold respectively (Figure 5d). The simulations also indicate that the density of osteoblast precursors (OB_p_), active osteoblasts (OB_a_) and active osteoclasts (OC_a_) increase 2.14-fold, 1.65-fold and 1.97-fold respectively (Figure 5a-c) while bone volume decreases to 91% at the end of year-5 (Figure 5e), which are qualitatively consistent with clinical observations [67]. The synergistic effect of TGF-β and MM-BMSC adhesion on IL-6 production by BMSCs is also observed. The simulated ratio of IL-6 production by two ligands stimulation compared to the sum of each ligand separately is between 1.48-fold and 2.19-fold, while the reported experimental ratio is between 1.45-fold and 2-fold [35].

The quantitative and qualitative agreements between our simulations and clinical observations suggest that this proposed MM-bone model is able to capture some of the major features of the MM disease progression and so within the limitations of the model, appropriately reflect the MM disease progression in bone marrow.

## Discussion

### 4.1 The relative importance of regulation by the positive feedback cycles

There are two positive feedback cycles identified in our MM-bone model (see Figure 2). Based on the similarity of the clinically observed normalized increase in MM cells and the normalized decrease in bone volume and the model results (see Figure 5d-e), this suggests that the two feedback cycles included in the model are sufficient to jointly drive the disease interaction between MM cells and the bone microenvironment. However, the relative importance of the two positive feedback cycles is not yet clear. This issue can be addressed by performing a quantitative analysis on the positive feedback cycles.

Our quantitative analysis on the relative importance of the positive feedback cycles is based on comparing changes of variables when both positive feedback cycles are intact, with model outcomes when either one or other, or both, of the positive feedback cycles are disabled (i.e. blocked). The density of MM cells and the bone volume are suitable variables to track for evaluating the significance of the two positive feedback cycles on MM disease progression. The dynamic changes of MM-cell density and bone volume during MM disease progression may be quantified by calculating the ‘area under the curve’ (AUC), which is defined as the time integral of the change in the variable from beginning of the simulation to the end of the simulation. We propose that if the total change of MM-cell density is reduced by ten to fifty percent, or total change of bone volume loss is reduced by ten to fifty percent, when either one of the positive feedback cycles is blocked, then this feedback cycle is deemed ‘significant’ with respect to bone volume or MM-cell density.

The expression ‘vicious cycle’ is commonly used in the biological/cancer literature to identify positive feedback loops between the cancer cells and their microenvironment; however it is not usually given a quantitative definition. Here we propose that if blocking a positive feedback cycle is effective in more than halving the MM-cell density (i.e. greater than a fifty percent reduction) or more than halving the bone loss (i.e. greater than fifty percent reduction) or doing so to both, then we say this positive feedback cycle is a ‘vicious cycle’. This definition at least accords with the original intention of use of this expression by Mundy [13].

**Figure 7:**
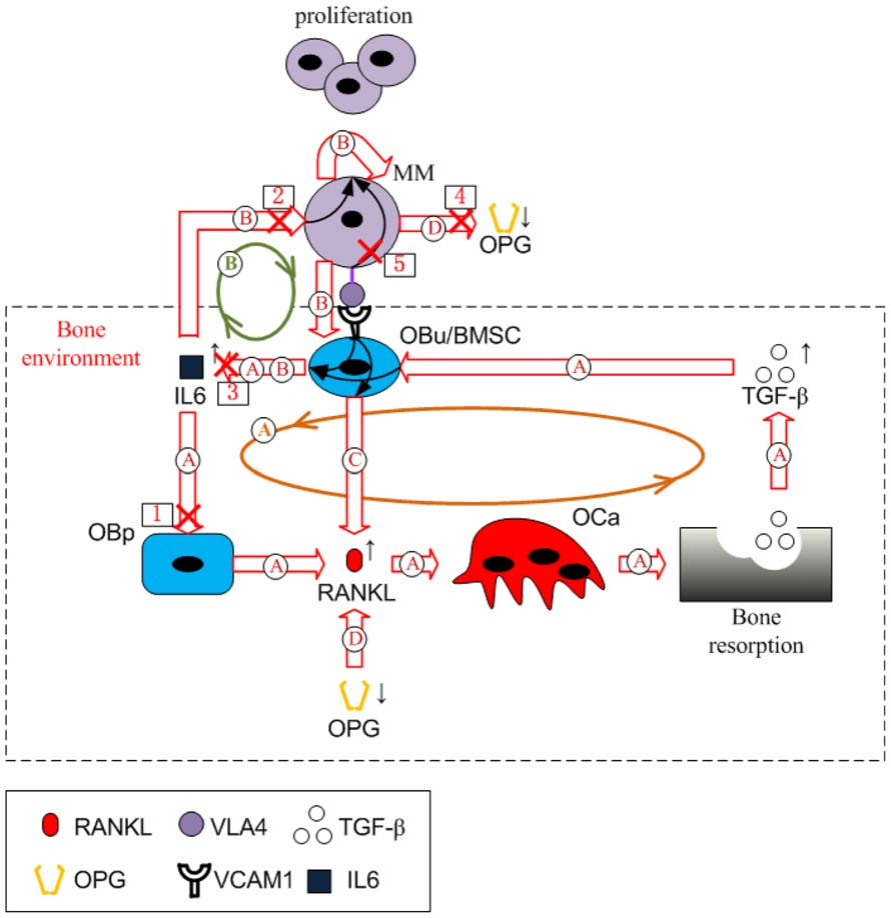
Schematic showing blocks in the MM-bone positive feedback cycles at specific points in the MM-bone model.

We investigated five cases where the two positive feedback cycles are blocked at different points in their cycle, namely (i) positive feedback cycle A is blocked at the point of IL-6 interaction with osteoblast precursors, (ii) positive feedback cycle B is blocked at the point of IL-6 interaction with MM cells, (iii) positive feedback cycle A and B are simultaneously blocked for IL-6 at the point of IL-6 production, (iv) an additional contribution to positive feedback cycle A is blocked at the point of OPG degradation by MM cells, and (v) an additional contribution to positive feedback cycle B is blocked at the point of MM-BMSC adhesion-stimulated MM cell proliferation. The precise blocking points of all these cases are illustrated in Figure 7.

**Figure 8:**
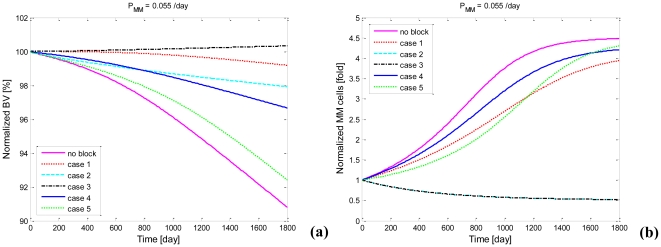
Model outputs after blocking positive feedback cycles in the MM-bone model. (a) Bone volume after blocking positive feedback cycles at specified points. (b) The density of MM cells after blocking positive feedback cycles at specified points. Case 1 Positive feedback cycle A is blocked at the point of interaction of IL-6 and osteoblast precursors. Case 2 Positive feedback cycle B is blocked at the point of interaction between IL-6 and MM cells. Case 3 Positive feedback cycles A and B are simultaneously blocked at the point of IL-6 production by BMSC. Case 4 Positive feedback cycle A is blocked at the point of OPG degradation by MM cells. Case 5 Positive feedback cycle B is blocked at the point of MM-BMSC adhesion-stimulated MM-cell proliferation.

Figure 8a-b show dynamic changes of bone volume and MM-cell density corresponding to each of the above-mentioned cases. It is noted here that *P_MM_* is selected as 0.055/day for the purpose of clearly displaying the results, but in fact, the conclusions for this value hold true for other values of *P_MM_*. A marked reduction of bone volume loss is observed in the first and the second case, while there is a slight increase in bone volume observed in the third case. Slightly less density of MM cells is observed in the first case whereas a marked decrease in MM-cell density is observed in the second and the third case (curves of the second and the third case are overlapped in the Figure 8b).

These changes suggest to us that MM cell population growth and bone volume loss are reversed when these two positive feedback cycles are simultaneously blocked, while they are partly inhibited when only one of the positive feedback cycles is blocked. Furthermore as shown in Table 7, the AUC of bone volume when the positive feedback cycle A and B are blocked compared to that when both positive feedback cycles are intact, expressed as a percentage is 6.74% and 31.06% respectively. The AUC of MM-cell density when the positive feedback cycle A is blocked compared to that when both positive feedback cycles are intact, expressed as a percentage is 79.77%. Based on these quantitative results for bone volume and MM-cell density changes and our definition, IL-6 stimulated RANKL production by osteoblast precursors is deemed to be significant with respect to bone volume, and IL-6 stimulated MM cell proliferation is deemed significant with respect to both bone volume and MM-cell density. Additionally, both positive feedback cycles would qualify as ‘vicious cycles’ with respect to bone volume changes. In contrast, positive feedback cycle A is deemed not to be a ‘vicious cycle’ with respect to MM-cell density, while positive feedback cycle B is deemed to be a ‘vicious cycle’ with respect to MM-cell density.

**Table 7:** The percentages of AUC of bone volume and MM-cell density when positive feedback cycles are blocked to those when these cycles are intact.

	Case 1	Case 2	Case 3	Case 4	Case5
Percentage of AUC of bone volume	6.74%	31.06%	↑	38.47%	78.35%
Percentage of AUC of MM-cell density	79.77%	↓	↓	89.76%	79.4%

Note: case 1: Positive feedback cycle A is blocked at regulation mechanism between IL-6 and osteoblast precursors; case 2: Positive feedback cycle B is blocked at regulation mechanism between IL-6 and MM cells; case 3: Positive feedback cycle A and B are simultaneously blocked at regulation mechanism of IL-6 production; Case 4: additional pathway to positive feedback cycle A is blocked at regulation mechanism of OPG degradation by MM cells. Case5: additional pathway to positive feedback cycle B is blocked at regulation mechanism of MM-BMSC adhesion-stimulated MM-cell proliferation.

In addition, a marked reduction of bone volume loss is observed in the fourth case, while slightly lesser density of MM cells is observed in the fourth case. As shown in Table 7, the AUC of bone volume in the fourth case compared to that when positive feedback cycles are intact, expressed as a percentage is 38.47%, while the AUC of MM-cell density in the fourth case compared to that when positive feedback cycles are intact, expressed as a percentage is 89.76%. These changes suggest to us that degradation of OPG by MM cells has a very significant impact on bone volume loss, while it almost has no impact on MM cell population growth. In terms of the fifth case, both a slight reduction of bone volume loss and slightly lesser density of MM cells are observed. The percentage of AUC of bone volume and the percentage of AUC of MM-cell density in the fifth case, compared to those when both positive feedback cycles are intact are 78.35% and 79.4% respectively (see Table 7), indicating that MM-BMSC adhesion-stimulated MM cell proliferation has neither a significant impact on the bone volume loss nor on MM cell population growth.

**Figure 9:**
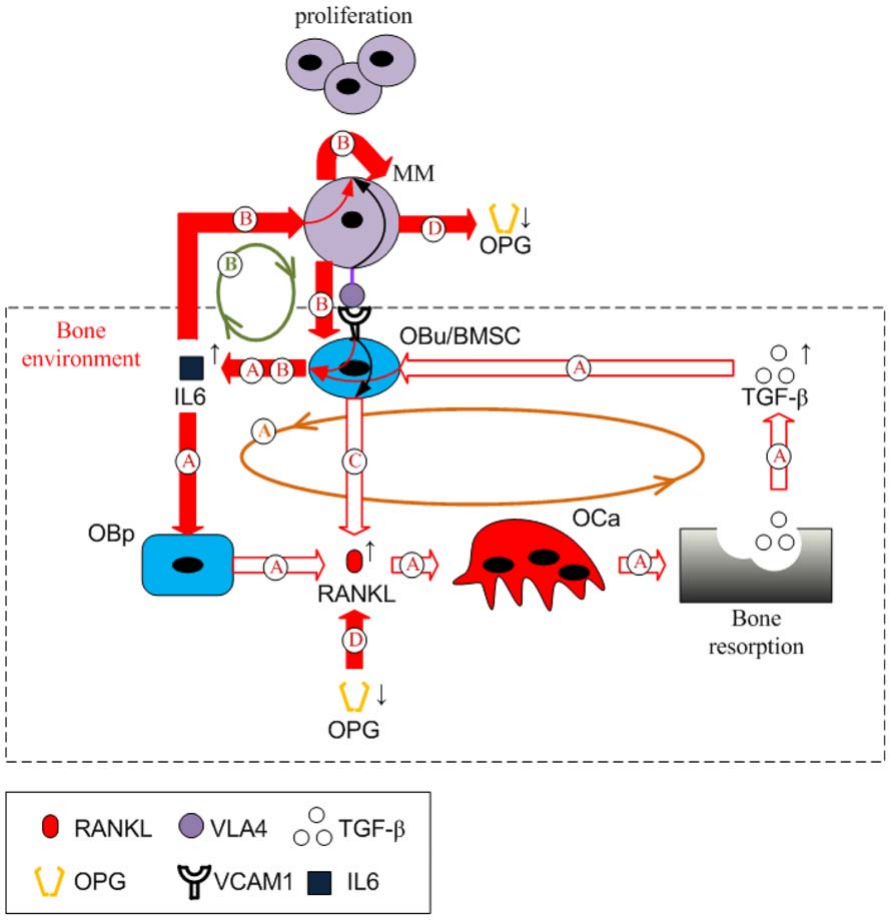
Schematic of dominant regulation points in the MM-bone positive feedback cycles. Filled red arrows highlight the dominant regulation points in the positive feedback cycles.

From these analyses, a picture of the dominant processes emerges. In the presence of MM cells, MM-BMSC adhesion and TGF-β induce ‘synergistic’ production of IL-6 by BMSCs. The substantially increased IL-6 concentration stimulates proliferation of MM cells leading to enhanced MM-BMSC adhesion and more degradation of OPG. On the other hand, increased RANKL production by osteoblast precursors together with decreased OPG concentration result in more bone resorption and more TGF-β released from bone matrix. The dominant processes in the two positive feedback cycles are highlighted by the red arrows in Figure 9. These dominant processes suggest potential drug targets to achieve different therapeutic objectives. For example, targeting IL-6-stimulated RANKL production by osteoblast precursors can reduce bone loss and so improve MM-induced bone lesions, while targeting IL-6-stimulated MM cell proliferation can reduce MM tumor burden and contribute to improving MM-induced bone lesions.

### Conclusion

In this paper, we have developed a computational model describing interactions between multiple myeloma and bone. This computational model is based on the previous bone remodeling model of Pivonka et al. [1], and explicitly considers IL-6 and MM-BMSC adhesion related pathways. Inclusion of these new pathways leads to the formation of two positive feedback cycles in this model. The parameters of this model are estimated based on reported values in the literature, and when required, best-fit parameter estimates are made using a least-square optimization criterion. Using this approach, the progression of MM disease is simulated numerically. Our model simulations are qualitatively and quantitatively consistent with known clinical observations for both normal bone physiology and for MM disease progression. This model suggests that the two MM-bone positive feedback cycles employed in this computational model are sufficient to jointly drive MM disease progression.

Analysis of the model behavior resulted in the clarification of the relative importance of the two positive feedback cycles, and identified the dominant influences within the feedback cycles. The dominant influences contributing to the feedback cycles suggest possible drug targets, which are different for different clinical objectives.

It is hoped that this computational model describing the interactions between multiple myeloma and bone can be improved over time, and eventually applied as a modeling platform for analyzing the relative efficacy of various therapeutic interventions.

## Supporting Information

Supporting Information S1 (DOC)
