# Supplementary material for: Computational Modeling of Interactions between Multiple Myeloma and the Bone Microenvironment
Source: PLoS One. 2011 Nov 8;6(11):e27494. doi: 10.1371/journal.pone.0027494 (PMC3210790; doi:10.1371/journal.pone.0027494)
Supplement: Supporting Information S1 — (DOC) [file pone.0027494.s001.doc]

**Supporting Information for “Computational Modeling of Interactions between Multiple Myeloma and the Bone Microenvironment”**

**Appendix A**

According to Eq.(1) the ‘activator’ function of MM cell proliferation in response to MM-BMSC adhesion (through VCAM-1 binding to VLA-4) is as follows:

$$\pi_{act,RANKL}^{VCAM1}=\frac{VCAM1}{VCAM1+K_{M,VCAM1,MM,act}}$$

The VCAM-1 binding to VLA-4 is described by the following reaction:

where *k_f_* and *k_b_* are the forward and backward rates of reaction respectively. According to mass action kinetics [1], the binding of VCAM-1 binding to VLA-4 at the steady state is given by:

$$VACM1\_VLA4=K_{A,VCAM1}\cdot VCAM1\cdot VLA4$$

Where, *VCAM1_VLA4* represents complex of VCAM-1 binding to VLA-4 and *K_A,VCAM1_* (= *k_f_* / *k_b_*) is association rate constant of VCAM-1 binding to VLA-4.

As we assume that the number of BMSC is constant and VCAM-1 expression on the surface of BMSC is constant, the total concentration of VCAM-1 (including free and bound) is constant and so we have:

$$VCAM1\_VLA4=VCAM1_{tot}-VCAM1$$

By inserting Eq. into Eq., VCAM-1 is solved as follows:

$$VCAM1=\frac{VCAM_{tot}}{1+K_{A,VCAM1}\cdot VLA4}$$

Inserting Eq. into Eq., we obtain the ‘activator’ function $\pi_{act,MM}^{VCAM1}$.

$$\pi_{act,MM}^{VCAM1}=\frac{1}{1+\frac{(1+VLA4\cdot K_{A,VCAM1})\cdot K_{M,VCAM1,MM,act}}{VCAM1_{tot}}}$$

Eq. shows that $\pi_{act,MM}^{VCAM1}$ decreases with the increase in VLA-4 concentration, which is consistent with our expectation. Because VLA-4 increases with an increase in density of MM cells while VCAM-1 is constant, the increase in the complex *VCAM1_VLA4* is less than the increase in free VLA-4, leading to decrease in occupancy of VLA-4 on the surface of MM cells and so decrease in $\pi_{act,MM}^{VCAM1}$.

**Appendix B**

To calculate the concentration of PTH, let

$$PTH=\frac{P_{PTH,d}+\beta_{PTH}}{D_{PTH}}$$

where, *P_PTH,d_* is the external production rate of PTH with the unit of $pM\cdot day^{-1}$. *β_PTH_* is the endogenous production rate of PTH with the unit of $pM\cdot day^{-1}$. *D_PTH_* is the degradation of PTH. To calculate the concentration of TGF-β let,

$$TGF\beta=\frac{P_{TGF\beta,d}+\alpha\cdot k_{res}\cdot OC_{a}}{D_{TGF\beta}}$$

where, *P_TGFβ,d_* is the external production rate of PTH with the unit of $pM\cdot day^{-1}$. *α* is a proportionally constant expressing the TGF-β content stored in the bone volume with the unit of $pM\cdot\%^{-1}$, and *k_res_* is the relative rate of bone resorption by active osteoclasts with the unit of $\%\cdot pM^{-1}\cdot day^{-1}$. *D_TGFβ_* is the degradation of TGF-β.

These calculations for concentrations of PTH and TGF-β are the same as described in the [2].

**References**

1. Lauffenburger DA & Linderman JJ (1993) Receptors: Models for Binding, Trafficking, and Signaling. Oxford University Press, Inc.

2. Pivonka P, Zimak J, Smith DW, Gardiner BS, Dunstan CR, et al. (2008) Model structure and control of bone remodeling: A theoretical study. Bone 43**:** 249-263.
